# Comparing the efficacy of vaginal micronized progesterone gel and capsule for prevention of preterm birth in singleton pregnancies with short cervical length at midtrimester: an indirect comparison meta-analysis

**DOI:** 10.3389/fphar.2023.1153013

**Published:** 2023-07-12

**Authors:** Doron Kabiri, Yael Hamou, Gali Gordon, Yosef Ezra, Ilan Matok

**Affiliations:** ^1^ Department of Obstetrics and Gynecology, Hadassah Hebrew Medical Center and Faculty of Medicine, Hebrew University of Jerusalem, Jerusalem, Israel; ^2^ The Division of Clinical Pharmacy, School of Pharmacy, Institute for Drug Research, The Hebrew University of Jerusalem, Jerusalem, Israel

**Keywords:** vaginal micronized progesterone, preterm birth prevention, short cervical length, progesterone gel, progesterone capsule

## Abstract

**Objective:** To evaluate the effectiveness of vaginal progesterone in preventing preterm birth in women with a singleton gestation and short cervical length and to determine which of the two formulations, micronized progesterone vaginal capsule versus vaginal gel containing micronized progesterone, is more effective for preventing preterm birth.

**Data sources:** A systematic search was performed in the following databases: EMBASE, PubMed (MEDLINE), The Cochrane Library, and the Clinical Trials Registry (clinicaltrials.gov).

**Study eligibility criteria:** Randomized controlled trials (RCTs), prospective and retrospective observational studies were included. We searched for progesterone administration to prevent preterm birth in asymptomatic women with a shortened cervix (<25 mm) measured by ultrasound in the second trimester of singleton pregnancy.

**Study appraisal and synthesis methods:** Assessments of the risk of bias of RCTs were performed by applying the Cochrane Collaboration’s Risk of Bias Tool; non-randomized control trials were evaluated with the Newcastle–Ottawa Scale (NOS). The primary outcome was preterm birth ≤33 weeks of gestation. Pooled relative risks (RR) and 95% CI’s were calculated for dichotomous outcomes. Heterogeneity of treatment effect was assessed with the I^2^ statistic. We pooled results of the primary outcome for individual studies using a random-effect model. We then performed a network meta-analysis to pool indirect comparisons between the two formulations (gel vs capsule). This analysis was performed using the network meta-analysis package within the R environment.

**Results:** Five studies met the inclusion criteria (4 RCTs, one cohort study) including 1,048 women. The meta-analysis demonstrated that vaginal micronized progesterone significantly reduces preterm birth risk, Risk Ratio = 0.63; 95% CI, 0.48–0.82; *p* = 0.0006; with no heterogeneity between the studies: *I* ^2^ = 0%. In the network meta-analysis, no significant difference was demonstrated (OR = 0.85; 95% CI, 0.43–1.69) between the effect of the two formulations of vaginal micronized progesterone (vaginal gel versus vaginal capsules) on the risk of PTB.

**Conclusion:** Vaginal progesterone is associated with a decreased risk of premature birth in women with a shortened cervix in the second trimester of pregnancy. No differences were found between vaginal micronized progesterone in gel or capsule formulations.

**Systematic Review Registration:** PROSPERO, identifier CRD42020165198.

## Introduction

Prematurity is the leading cause of death in children under 5 years of age. The prevalence of premature births varies from 5% to 18%, with approximately 15 million babies being born preterm every year. Prematurity is associated with short-term and long-term complications (Organization, 2015). The higher morbidity rate of premature infants as compared to term neonates (up to 7 times higher) may require unique treatments and hospital admission for weeks or months at the neonatal intensive care unit (NICU). The most common causes of this morbidity rate include hypothermia, hypoglycemia, respiratory distress, newborn infections, neonatal jaundice, and feeding difficulties. Complication severity increases with decreasing gestational age and birth weight, especially in babies born before 28 weeks and weighing less than 1,500 g.

Cervical shortening, particularly between 14 and 28 weeks of gestation, is associated with an increased risk for spontaneous preterm birth. A short cervix is generally defined as a transvaginal sonographic cervical length (CL) of 25 mm or less in the mid-trimester of pregnancy. An inverse relationship exists between the cervical length measured by ultrasonography during pregnancy and preterm delivery frequency (American College of O et al., 2012; American College of and Gynecologists' Committee on Practice, 2021; Iams et al., 1996; Taipale and Hiilesmaa, 1998).

Progesterone increases the arterial blood supply to the fetus and glycogen production and prevents bacteria from entering the uterus by thickening the cervix. Its role in the second and third trimesters of pregnancy is less clear. However, its effects on the uterus, myometrium and cervix explain the logic of using it to prevent spontaneous preterm birth. Progesterone exerts an inhibitory effect on the expression of contraction-associated protein genes in the myometrium and hinders the production of stimulatory prostaglandins (PG). Cervical remodeling (softens, ripens and dilates) occurs with loss of progesterone’s tissue effects, usually starting several days or weeks before the onset of regular uterine contractions, a late step in the parturition process. Decreased local progesterone responsiveness, termed “functional withdrawal,” probably instigates cervical remodeling (Sfakianaki and Norwitz, 2006). The interval between cervical ripening and labor is when treatment can prolong the pregnancy and prevent premature birth.

Interventions to reduce the risk of preterm birth in cases of cervical shortening, including progesterone, cervical cerclage, and cervical pessary, have been extensively investigated as treatment options for women with varying risk factors. Guidelines have been developed, matched to the most effective treatment for each case.

The present review and meta-analysis will focus on the management of asymptomatic women with a singleton gestation with short cervical length at mid-trimester, and the comparative effectiveness of vaginal micronized progesterone delivered via gel or capsule.

### Vaginal micronized progesterone

Natural micronized progesterone is chemically identical to ovarian progesterone. Its micronization decreases particle size, increases surface area, improves absorption and bioavailability, and decreases metabolic and vascular side effects. The advantage of vaginal progesterone is its high uterine bioavailability since uterine exposure occurs before the liver’s first-pass effect. The term ‘first uterine pass effect’ refers to the phenomenon where progestational effects on the endometrium persist, even in the presence of low plasma levels (Balasch et al., 1996; Bulletti et al., 1997; De Ziegler et al., 1997). In addition to the aforementioned advantages, the vaginal route of administration is considered superior to oil-based intramuscular (IM) injections, which are often associated with pain and discomfort. The oral route is characterized by low absorption and higher doses.

In 2018, a meta-analysis of patient-level data from randomized clinical trials was performed to determine whether vaginal progesterone administration prevents preterm birth and improves neonatal outcomes in asymptomatic women with a singleton gestation with a short cervix. The analysis comprised of 974 women (498 received vaginal progesterone and 476 received placebo), who had a cervical length ≤25 mm and were enrolled in five high-quality clinical trials. The results demonstrated that vaginal progesterone administration was significantly associated with a reduced risk of preterm birth before 33 weeks of gestation [relative risk, 0.62; 95% confidence interval (CI), 0.47–0.81; *p* = 0.0006]. Moreover, the administration of vaginal progesterone resulted in improvements in several neonatal outcomes, including respiratory distress syndrome, composite neonatal morbidity and mortality, birthweight <1500 and <2500 g, and admission to the neonatal intensive care unit, with relative risks ranging from 0.47–0.82.

Progesterone treatment should be started between 16 and 24 weeks of gestation and continued until 36 weeks. It is recommended prophylactic for women who have one of the following: 1. A cervical length of 25 mm or less detected via transvaginal ultrasound scan performed between the 18th and 24th weeks of pregnancy, 2. A history of mid-trimester loss (from 16 weeks of pregnancy onwards) or spontaneous preterm birth (up to 34 weeks of pregnancy), or 3. Both a history of spontaneous preterm birth and a short cervix in the current pregnancy. (Excellence NIfHaC, 2015).

Natural or micronized progesterone is typically administered vaginally. Vaginal progesterone provides a notable advantage due to its high uterine bioavailability. This is attributed to the fact that the first pass effect through the liver is bypassed, allowing for direct delivery to the uterus (Balasch et al., 1996; Bulletti et al., 1997; De Ziegler et al., 1997). Two dosage forms could be used: 1.100 mg and 200 mg micronized vaginal progesterone capsules (Utrogestan); 2. A 1.125-g vaginal gel containing 90 mg micronized progesterone per dose (Crinone 8%).

## Objectives

We aim to re-examine progesterone’s effectiveness in vaginal administration in preventing preterm birth <33 weeks of gestation in women with singleton gestation with short cervical length and determine which of the two formulations, micronized progesterone vaginal capsule versus vaginal gel containing micronized progesterone, is more effective for the prevention of preterm birth. We hypothesized that the efficiency of the gel is superior to the efficiency of the capsule. A conclusion from this meta-analysis may help clinicians choose the best treatment for their patients.

## Methods

### Data sources

A systematic review and meta-analysis were performed according to the PRISMA guidelines (Preferred Reporting Items for Systematic Reviews and Meta-Analysis) (Moher et al., 2015) and was registered in the PROSPERO (approval number CRD42020165198). There was no need for Institutional Review Board approval as no patient-level information was included.

A systematic search was performed in the following databases: EMBASE, PubMed (MEDLINE), The Cochrane Library, and the Clinical Trials Registry (clinicaltrials.gov). The search was conducted without language or date restrictions. We included randomized controlled trials (RCT’s), prospective and retrospective observational studies, and case-control studies. We excluded reviews, meta-analyses, case reports, cross-sectional studies, guidelines, expert opinion, editorials, letters to the author, and comments. The search was performed with the EMBASE database in January 2020, using the Emtree method. It is a more comprehensive search and includes MeSH terms and their synonyms: progesterone, uterine cervix, premature labor. The whole word string is shown in the (Supplementary Figure S1).

We searched for treatment of vaginal micronized progesterone administration in asymptomatic women with a shortened cervix (<25 mm) measured by ultrasound in the second trimester of singleton pregnancy, to prevent preterm birth. Short cervical length was defined as below 25 mm measured in the second trimester of pregnancy, between 14 and 28 weeks of gestation. Studies in which a cervical length >25 mm was defined as a short cervix were not included.

The intervention group included any progesterone dose in either capsule, tablet, or gel, and the control group included placebo or any other non-pharmacological treatment, such as bed rest. We did not include studies in which the intervention group or the control group used progesterone that is not micronized progesterone or a different form of administration, for example, oral or IM, or another technology, for example, pessary or cerclage. Studies that included multiple gestations were excluded unless a separate analysis was performed for singleton pregnancies.

The primary outcome was preterm birth (PTB) before week 33 of gestation.

### Data extraction

Data extraction and data analysis were performed simultaneously, and independently by two reviewers (YH and GG). All studies that met inclusion criteria were assessed by publication titles, abstracts, and finally, full-text articles. Disagreements were resolved by discussion until reaching an agreement between the two reviewers or by referral to a third reviewer (DK). The following data were extracted from each study: authors, date of publication, study type, mean follow-up time, number of participants, intervention (dosage, vehicle, and treatment duration), and outcome details.

### Risk of bias assessment

Assessments of the risk of bias of RCTs were performed independently by two investigators (YH and GG) applying the Cochrane Collaboration’s Risk of Bias Tool (Review Manager (RevMan) Version 5.2.3. Copenhagen), which includes seven domains: random sequence generation (selection bias), allocation concealment (selection bias), blinding of participants and personnel (performance bias), blinding of outcome assessment (detection bias), incomplete outcome data (attrition bias), selective reporting (reporting bias), other bias (set for conflict of interest). This tool classifies study quality by risk of bias, (i.e., low, unclear, and high). For non-randomized control trials, we used the Newcastle–Ottawa scale (NOS). The NOS tool is based on eight criteria, and its grading ranges from 0 stars (the lowest quality) to 9 stars (the highest quality). A relevant study was required to reach at least a 5-star level in this system.

### Statistical analysis

Five studies met the inclusion criteria. Outcomes were analyzed on an intention-to-treat basis. The data synthesis was performed using the comprehensive meta-analysis software (CMA) version 3 (CMA v. 3, Biostat, Englewood, NJ). Different trials were combined to calculate pooled relative risk (RR) with 95% CI for dichotomous outcomes. Heterogeneity of treatment effect was assessed with the *I* ^2^ statistic. We pooled results of the primary outcome for individual studies using a random-effect model since there was a clinical variability between the studies (i.e., different populations, countries, formulations, dosages). However, heterogeneity was not found. We then performed a network meta-analysis to pool indirect comparisons between the two formulations (gel vs capsule). This analysis was performed using the package network meta-analysis within the R environment.

## Results study selection

**FIGURE 1:**
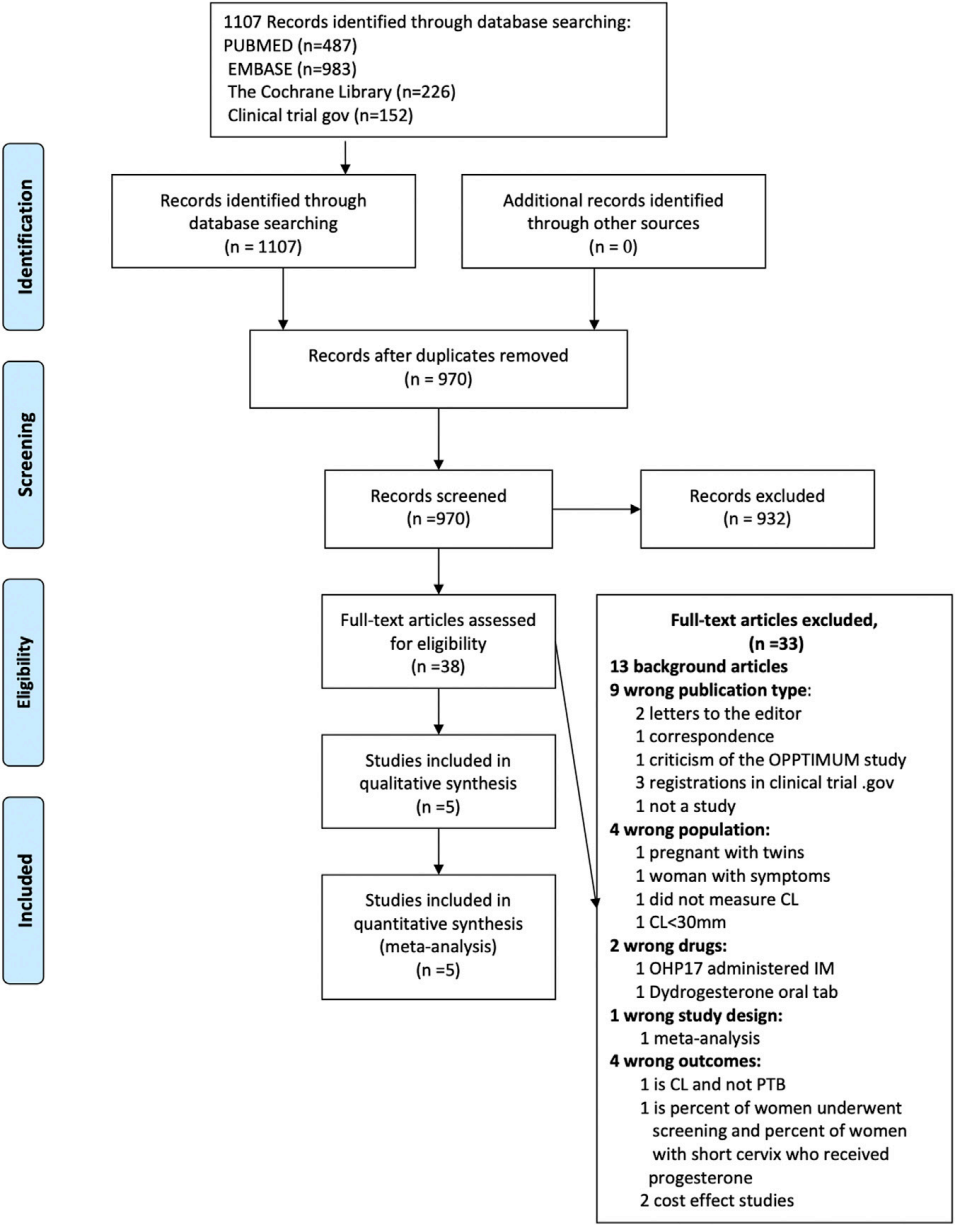
Publication selection and search process: The detailed process of selecting the including/excluding studies.

The systematic search yielded 1107 records: 137 were identified as duplicates, leaving 970 relevant records. Initial screening left 74 potentially relevant records. After the exclusion of irrelevant abstracts, 38 articles were selected for full-text evaluation. Thirty-three articles were excluded because they did not meet the criteria of publication type 9); population 4); drug 2); study design 1); outcomes 4); and 13 were background articles. Five studies met the inclusion criteria(DeFranco et al., 2007; Fonseca et al., 2007; Hassan et al., 2011; Norman et al., 2016; Maerdan et al., 2017) The detailed process of selecting the including/excluding studies is shown in Figure 1.

### Study characteristics

The characteristics of the included studies (DeFranco et al., 2007; Fonseca et al., 2007; Hassan et al., 2011; Norman et al., 2016; Maerdan et al., 2017) are listed in Table 1. Five studies and a total of 1,048 patients were included in the final analysis: four multicenter randomized control trials, conducted in hospitals from several countries, and one cohort study conducted at the Hospital of Peking University in China.

**TABLE 1:** Characteristics of included studies.

First author and publication year	Study design	Follow up	Number of subjects (treatment/control)	Age Mean (SD) Or Median (range)	0ervical length	Treatment group	Comparator group	Primary outcome
Hassan et al. 2011 (27)	RCT	between 19 + 0 and 23 + 6 weeks until 36+6 weeks	458 (235/223)	Treatment group	10–20 mm	8 percent vaginal gel containing 90 mg micronized progesterone per dose administered once daily in the morning	placebo gel	PTB <33 weeks
				26.5 (5.8)				
				____________				
				Comparator				
				26.2 (5.1)				
Eduardo B. Fonseca et al. 2007 (29)	RCT	: from 24 to 33+6 weeks	226 (114/112)	Treatment	<15 mm	200-mg capsules of micronized progesterone (Utrogestan, Besins International Belgium) every night	identical-appearing capsules of placebo containing safflower oil (Medicaps)	PTB <34 weeks
				29 (24–34)				
				____________				
				Comparator				
				29 (24–34)				
E. A. DeFranco et al. 2007 (31)	RCT	between 18 + 0 and 22 + 6 weeks until 37 + 0 weeks	31 (12/19)^a^	Treatment group	<25 mm^a^	8 percent vaginal gel containing 90 mg micronized progesterone per dose administered once daily in the morning	placebo gel	PTB <32 weeks
				27 (4.9)				
				____________				
				Comparator				
				26.2 (5.1)				
Jane Elizabeth Norman et al. 2016 (28)	RCT	34 weeks of gestation, during labour and delivery, during the neonatal stay and at 1 and 2 years post-delivery	251 (133/118)	Treatment^c^ group	≤25 mm	progesterone 200 mg soft capsules daily at bedtime	placebo	PTB <34 weeks
				31.4 (5.8)				
				____________				
				Comparator				
				31.5 (5.6)				
Malipati Maerdan et al. 2017 (30)	cohort	20–24 weeks of gestation until delivery	82 (40/42)	Treatment group	10 < cl < 25 mm	progesterone capsules 200 mg each night	bed rest -simply resting activity restriction	PTB <33 weeks
				31 (29–34)				
				____________				
				Comparator group				
				31 (29–35)				

^a^ Was taken from the meta-analysis of IPD.

^b^ (DeFranco reported outcome for cervical length< 28 mm).

^c^ This average is of all the women who participated in the study and not just of those with the shortened cervix.

Hassan et al. (2011) 2011 and Maerdan et al. (2017) 2016 reported PTB< 33 weeks, Fonseca et al. (2007) 2007 and Norman et al. (2016) 2016 reported PTB <34 weeks. DeFranco et al. (2007) 2007 reported PTB <32 weeks of gestation. We completed the missing data for our primary outcome, PTB <33 for Fonseca et al. (2007) 2007, Norman et al. (2016) 2016., and DeFranco et al. (2007) 2007, from a meta-analysis of individual patient data published in 2018, in which these studies and outcomes were included(Romero et al., 2018) ^(25)^.

Of the 1,048 included participants, 534 were treated with vaginal micronized progesterone, 247 women in two studies received 8 percent vaginal gel containing 90 mg micronized progesterone per dose (DeFranco et al., 2007; Hassan et al., 2011), and 287 women in three studies received progesterone 200 mg soft capsules (Fonseca et al., 2007; Norman et al., 2016; Maerdan et al., 2017).

**FIGURE 2:**
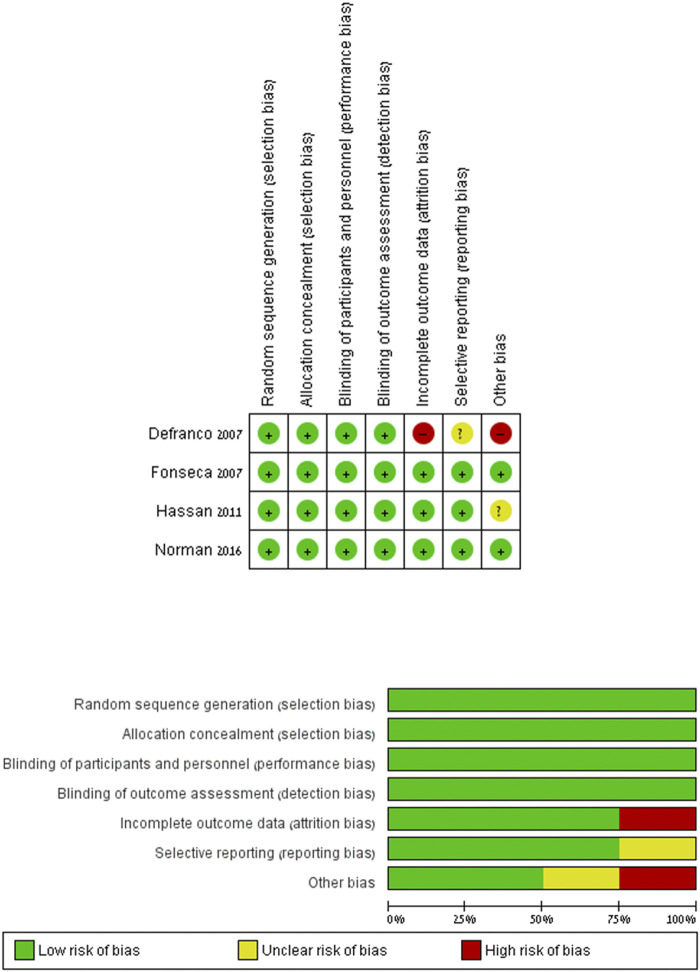
Risk of bias: the risk of bias in each included RCT study. Review Manager (RevMan) Version 5.2.3.

Figure 2 summarizes the risk of bias in each included RCT study. The risk of bias was low in all categories in the studies by Hassan et al., 2011). 2011^(32)^, Fonseca et al. (2007). 2007^(34)^, and Norman et al. (2016). 2016 ^(33)^. Regarding DeFranco et al. (2007). 2007 ^(36)^, the risk is unclear. After the randomization and the primary analysis (O'Brien et al., 2007), the investigators changed the original planned secondary analysis, which is the analysis relevant to our research. They intended to include women with short cervical length as a single risk factor and eventually expanded the research to women with prior preterm birth in addition to a shortened cervix. In addition, 48 women were lost to follow-up for the primary analysis. Table 2 summarizes the cohort study’s quality assessment of Maerdan et al. (2017). The article quality was rated as good, receiving eight stars in NOS.

**TABLE 2:** The Newcastle-Ottawa Scale quality assessment of the included cohort study

	Selection				Comparability	Outcome			Total (9 ⋆)
Study ID	Representativeness of exposed cohort (⋆)	Selection of non-exposed cohort (⋆)	Ascertainment of exposure (⋆)	Demonstration that outcome of interest was not present at the start of the study	(⋆⋆)	Assessment of outcome (⋆)	Was follow-up long enough for outcomes to occur	Adequacy of follow up (⋆)	
Maerden et al. (2017)	⋆	⋆	-	⋆	⋆⋆	⋆	⋆	⋆	**8**

Thresholds for converting the Newcastle-Ottawa scales to Agency for Healthcare Research and Quality standards (good, fair, and poor): Good quality: 3 or 4 stars in selection domain AND 1 or 2 stars in comparability domain AND 2 or 3 stars in outcome/exposure domain, Fair quality: 2 stars in selection domain AND 1 or 2 stars in comparability domain AND 2 or 3 stars in outcome/exposure domain, Poor quality: 0 or 1 star in selection domain OR 0 stars in comparability domain OR 0 or 1 stars in outcome/exposure domain. The article quality was rated as good, receiving eight stars in NOS.

### Meta-analysis

**FIGURE 3:**
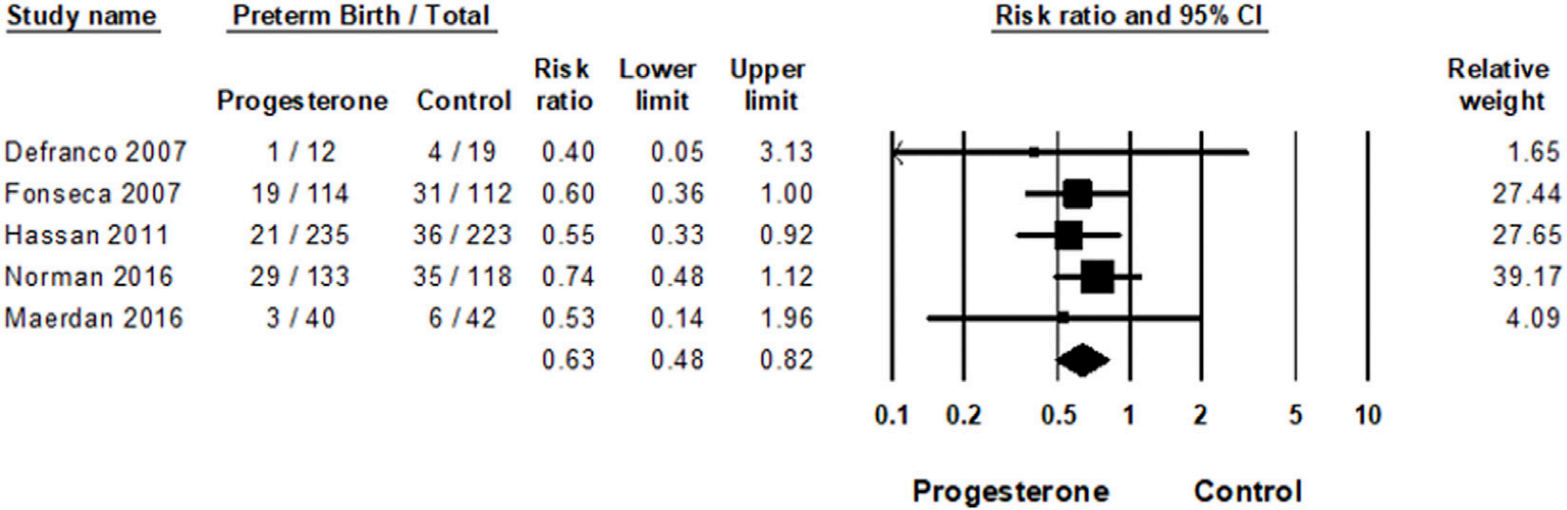
Meta-analysis of the effect of vaginal progesterone on the risk of preterm birth. Forest plot for the effect of vaginal micronized progesterone on the risk of PTB<33 weeks of gestation; Heterogeneity: *I* ^2^ = 0%; *Z* = 3.42; *p* = 0.001.

As shown in Figure 3 and corresponding to the previous meta-analysis (Romero et al., 2018), vaginal micronized progesterone significantly reduced preterm birth risk before 33 weeks of gestation, risk ratio = 0.63; 95% CI, 0.48–0.82; *p* = 0.0006; with no heterogeneity between the studies: *I* ^2^ = 0%.

### Network meta-analysis

**FIGURE 4:**
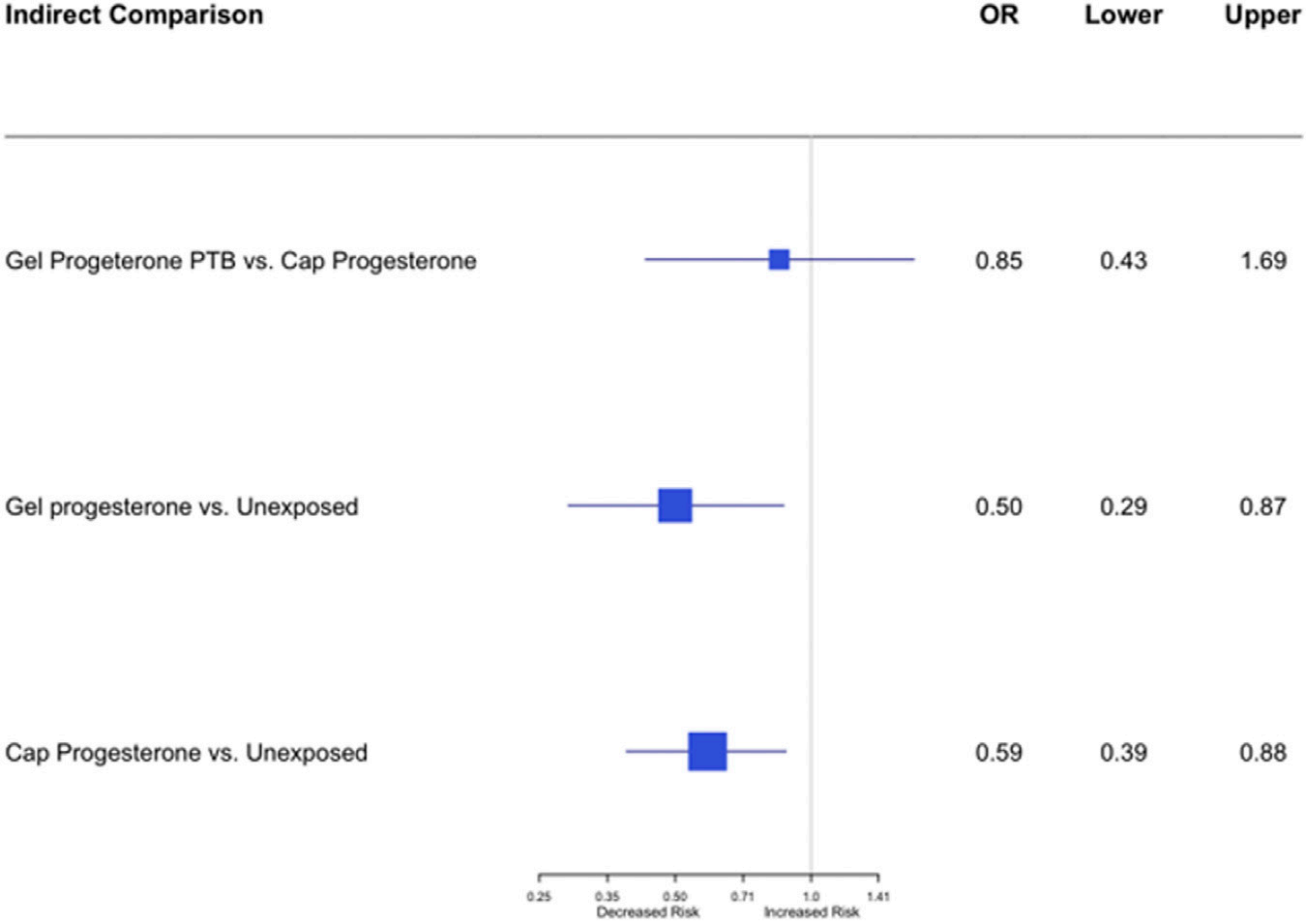
Network indirect meta-analysis: an indirect comparison between the effect of vaginal gel versus vaginal capsules of micronized progesterone on the risk of PTB<33 weeks of gestation; OR = 0.85; 95% CI, 0.43–1.69.

In the network meta-analysis, an indirect comparison was made between the effect of the two formulations of vaginal micronized progesterone (vaginal gel versus vaginal capsules) on the risk of PTB<33 weeks of gestation. As shown in Figure 4, no statistically significant difference was demonstrated (OR = 0.85; 95% CI, 0.43–1.69).

### Main findings

This systematic review and meta-analysis showed that vaginal micronized progesterone significantly reduces the risk of preterm birth before 33 weeks of gestation in women with a singleton gestation with short cervical length in the mid-trimester. No significant difference was found in the network meta-analysis comparing the effect of two vaginal formulations of micronized progesterone (vaginal gel versus vaginal capsules) on the risk of PTB<33 weeks of gestation.

In our indirect comparison meta-analysis, we compared the effect of vaginal gel vs vaginal capsules on the risk of PTB<33 weeks of gestation, which had not been investigated before. We hypothesized that the gel would be more effective and have fewer side effects, as seen in some studies examining efficacy when used for different indications [e.g., luteal phase support in blastocyst stage embryo transfers (Wang et al., 2009)]. No significant differences were found. This may be because the comparison is indirect, and it is possible that if a head-to-head study were conducted in the future, differences in side effects might be detected. In addition, the dosages of the two formulations differ: the gel contains 90 mg, and the capsule contains 200 mg.

### Strengths and limitations

Our study has several strengths. First, the method of searching, extracting, collecting, and evaluating the data, analyzing and reporting the findings were conducted systematically according to PRISM guidelines. We included all available data without language restriction. Second, the included studies’ quality was evaluated, and all the studies were found to be of good quality. Third, we added another study to the latest meta-analysis conducted on the subject that, in our opinion, despite not being an extensive study and being methodologically inferior (cohort rather than RCT), is relevant and important as it includes information on a different ethnic group and risk factors, and thus expands the external validity of the study. Another strength lies in the absence of statistical heterogeneity. Therefore, it can be determined that the effect found is indeed due to intervention, vaginal progesterone, and not to any bias.

This meta-analysis also has limitations. The main limitation in this meta-analysis lies in the source of the data. As mentioned above, since in some studies the primary outcome (rate of preterm births), was determined for a different gestational week, it was necessary to take the outcome we extracted (PTB<33 weeks) from Romero’s meta-analysis of individual patient data (Romero et al., 2018). We assume that the results are accurate, but we have no way of determining this with certainty. We contacted the principal investigator of the requested studies and asked for this data but there was no response. Another limitation of the study is the lack of neonatal outcomes. Neonatal outcome data, including short-term prematurity complications (i.e., RDS, NEC, NICU, IVH), would have strengthened our results. However, these data were not reported in some of the studies, or not provided for the specific study group we defined (women with a single fetus and a shortened cervix) but rather all participants, including twins or other risk factors. We could not perform a publication bias analysis since there were 5 studies included in our systematic review and meta-analysis.

### Comparison with existing literature

The studies selected for our meta-analysis investigated the effect of progesterone on preterm birth in women with various risk factors, including asymptomatic women with shortened cervix at mid-trimester.

In 2007, DeFranco et al. (2007) demonstrated that in women with a short cervix (CL < 28 mm) and prior preterm birth, the rate of PTB ≤32 weeks of gestation was significantly lower for those receiving progesterone than it was for those receiving the placebo (0% vs 29.6%, *p* = 0.014). In the same year, Fonseca et al. (2007) found that among women with singleton pregnancies and cervical length ≤15 mm, the incidence of spontaneous PTB≤ 34 weeks of gestation was significantly higher in the placebo group than in the progesterone group (32.1% vs 17.5%; relative risk, 0.54; 95% CL, 0.34–0.88; *p* = 0.02). In 2011, Hassan et al. (2011) reported that in women with a short cervix (CL = 10–20 mm) in the mid-trimester, those allocated to receive progesterone had a significantly lower rate of PTB<33 weeks of gestation compared with those allocated to receive the placebo (8.9% vs 16.1%; relative risk, 0.54; 95% CL, 0.33–0.89; *p* = 0.01). Each study defined the primary outcome and number of preterm births at different gestational weeks. Romero et al. (2012) performed a systematic review and meta-analysis of individual patient data from the three studies mentioned above and two other studies that also included twin gestations. They aimed to determine whether the use of vaginal progesterone in asymptomatic women with singleton or twin gestations, with a sonographic short cervix (≤25 mm) in the mid-trimester, reduces preterm birth risk and improves neonatal morbidity and mortality. Among singleton gestations, the administration of vaginal progesterone was associated with a statistically significant reduction in preterm birth ≤33 (relative risk, 0.57; 95% CL, 0.40–0.81), ≤35 and ≤28 weeks of gestation; and also, a reduction in neonatal morbidity outcomes. In 2016, Norman et al. (2016) conducted the largest randomized trial of vaginal progesterone to prevent preterm birth in women at high risk due to several risk factors, including those with a short cervix. Progesterone had no significant effect on the primary outcome within the short cervix subgroups, PTB ≤34 weeks of gestation (odds ratio, 0.69; 95% CL, 0.39–1.2). Romero again performed a further meta-analysis of individual patient data that included Norman’s trial (Norman et al., 2016) in 2016 (Romero et al., 2016) and 2018 (Romero et al., 2018) and showed that vaginal progesterone administration was associated with a statistically significant reduction in the risk of PTB ≤34 weeks (relative risk, 0.66; 95% CL, 0.52–0.83 *p* = 0.0005) and PTB ≤33 weeks (relative risk, 0.62; 95% CL, 0.47–0.81 *p* = 0.0006) respectively. In 2016 Maerdan et al. (2017) performed a retrospective cohort study to determine the prevalence of short cervical length during the gestational period of 20–24 weeks in China. The study aimed to evaluate the efficacy of micronized progesterone in prolonging gestation among nulliparous patients with a short cervix. They found that the short cervical length (CL ≤ 25 mm) rate was less than expected (0.45%). They also found that compared to bed rest, the administration of progesterone in cervical length measurements ranging from 10 mm to 20 mm was significantly associated with a decrease in the occurrence of preterm birth before 33 weeks of gestation (9.5% versus 45.5%, *p* = 0.02). However, in cervical length between 20 mm and 25 mm there were no significant differences in their rate of spontaneous PTB<33 (5.3% versus 3.2%, *p* = 0.72). When the two groups’ data are combined (cervical length between 10 mm and 25 mm), the odds ratio for progesterone administration in prevention of PTB<33 weeks is OR = 0.53; 95% CL, 0.14–1.96 (Maerdan et al., 2017).

Using Romero’s meta-analysis data (Romero et al., 2016; Romero et al., 2018) and including Maerdan’s study (Maerdan et al., 2017), our meta-analysis reinforces the contribution of progesterone to preventing preterm birth in women with singleton gestation and a shortened cervix.

## Conclusions and implications

In this study, we aimed to re-examine progesterone’s effectiveness in preventing preterm birth and to determine which of the two formulations, micronized progesterone vaginal capsule versus vaginal gel containing micronized progesterone, is more effective. This network meta-analysis demonstrates that vaginal gel and vaginal capsule of micronized progesterone equally reduce the risk of preterm birth in women with a singleton gestation with short cervical length in the mid-trimester.

## Data Availability

The original contributions presented in the study are included in the article/Supplementary Material, further inquiries can be directed to the corresponding author.
