# Supplementary material for: Comparing the efficacy of vaginal micronized progesterone gel and capsule for prevention of preterm birth in singleton pregnancies with short cervical length at midtrimester: an indirect comparison meta-analysis
Source: Front Pharmacol. 2023 Jul 12;14:1153013. doi: 10.3389/fphar.2023.1153013 (PMC10368970; doi:10.3389/fphar.2023.1153013)
Supplement: Supplementary file 1 [file Table1.DOCX]

**Table 1: Characteristics of included studies.**

| First author and publication year | Study design | Follow up | Number of subjects (treatment/  control) | Age  Mean (SD)  Or Median(range) | 0ervical length | Treatment group | Comparator group | Primary outcome |
| --- | --- | --- | --- | --- | --- | --- | --- | --- |
| Hassan et al. 2011 (27) | RCT | between 19 + 0 and 23 + 6 weeks until 36+6 weeks | 458  (235/223) | Treatment group  26.5 (5.8)  ____________  Comparator group 26.2 (5.1) | 10-20 mm | 8 percent vaginal gel containing 90 mg micronized progesterone per dose administered once daily in the morning | placebo gel | PTB < 33 weeks |
| Eduardo B. Fonseca et al. 2007  (29) | RCT | : from 24 to 33+6 weeks | 226  (114/112) | Treatment group 29 (24-34)  ____________  Comparator group 29 (24-34) | <15mm | 200-mg capsules of micronized progesterone (Utrogestan, Besins International Belgium) every night | identical-appearing capsules of placebo containing safflower oil (Medicaps) | PTB < 34 weeks |
| E. A. DeFranco et al. 2007  (31) | RCT | between 18 + 0 and 22 + 6 weeks  until 37 + 0 weeks | 31  (12/19) ^*^ | Treatment group  27(4.9)  ____________  Comparator group 26.2 (5.1) | <25mm^*^ | 8 percent vaginal gel containing 90 mg micronized progesterone per dose administered once daily in the morning | placebo gel | PTB < 32 weeks |
| Jane Elizabeth Norman et al. 2016  (28) | RCT | 34 weeks of gestation, during labour and delivery, during the neonatal stay and at 1 and 2 years post-delivery | 251  (133/118) | Treatment^**^ group  31.4 (5.8)  ____________  Comparator group 31.5 (5.6) | ≤ 25mm | progesterone 200 mg soft capsules daily at bedtime | placebo | PTB < 34 weeks |
| Malipati Maerdan et al. 2017  (30) | cohort | 20-24 weeks of gestation until delivery | 82  (40/42) | Treatment group 31 (29-34)  ____________  Comparator group 31 (29-35) | 10 < cl < 25mm | progesterone capsules 200 mg each night | bed rest -simply resting activity restriction | PTB < 33 weeks |

*was taken from the meta-analysis of IPD ^(25)^ (DeFranco reported outcome for cervical length< 28mm).

** This average is of all the women who participated in the study and not just of those with the

shortened cervix.

| Study ID | selection | | | | comparability | outcome | | | Total  (9 ⋆) |
| --- | --- | --- | --- | --- | --- | --- | --- | --- | --- |
|  | Representativeness of exposed cohort (⋆) | Selection of non-exposed cohort (⋆) | Ascertainment of exposure (⋆) | Demonstration that outcome of interest was not present at the start of the study | (⋆⋆) | Assessment of outcome (⋆) | Was follow-up long enough for outcomes to occur | Adequacy of follow up (⋆) |  |
| **Maerden et al.**  **2016 ^(30)^** | ⋆ | ⋆ | - | ⋆ | ⋆⋆ | ⋆ | ⋆ | ⋆ | **8** |

**Table 2. The Newcastle-Ottawa Scale quality assessment of the included cohort study**

Thresholds for converting the Newcastle-Ottawa scales to Agency for Healthcare Research and Quality standards (good, fair, and poor): Good quality: 3 or 4 stars in selection domain AND 1 or 2 stars in comparability domain AND 2 or 3 stars in outcome/exposure domain, Fair quality: 2 stars in selection domain AND 1 or 2 stars in comparability domain AND 2 or 3 stars in outcome/exposure domain, Poor quality: 0 or 1 star in selection domain OR 0 stars in comparability domain OR 0 or 1 stars in outcome/exposure domain.
